# Oral fluid testing facilitates understanding of hepatitis A virus household transmission

**DOI:** 10.1017/S095026881900027X

**Published:** 2019-03-01

**Authors:** Becky Haywood, Richard S. Tedder, Kazim Beebeejaun, Koye Balogun, Sema Mandal, Nick Andrews, Siew Lin Ngui

**Affiliations:** 1 Blood Borne Virus Unit, National Infection Service, Public Health England, London, UK; 2 Department of Infectious Diseases, Faculty of Medicine, Imperial College London, London, UK; 3 Immunisation, Hepatitis and Blood Safety Department, National Infection Service, Public Health England, London, UK; 4 Department of Statistics and Modelling, National Infection Service, Public Health England, London, UK

**Keywords:** Hepatitis A, public health, non-invasive testing, oral fluid

## Abstract

The public health response to sporadic hepatitis A virus (HAV) infection, hepatitis A, can be complex especially when the index case is a child and no obvious source is identified. Identifying an infection source may avoid mass immunisation within schools when transmission is found to have occurred within the household. Screening of asymptomatic contacts via venepuncture can be challenging and unacceptable, as a result non-invasive methods may facilitate public health intervention. Enzyme-linked immunoassays were developed to detect HAV immunoglobulin M (IgM) and immunoglobulin G (IgG) in oral fluid (ORF). A validation panel of ORF samples from 30 confirmed acute HAV infections were all reactive for HAV IgM and IgG when tested. A panel of 40 ORF samples from persons known to have been uninfected were all unreactive. Two hundred and eighty household contacts of 72 index cases were screened by ORF to identify HAV transmission within the family and factors associated with household transmission. Almost half of households (35/72) revealed evidence of recent infection, which was significantly associated with the presence of children ⩽11 years of age (odds ratio 9.84, 95% confidence interval: 2.74–35.37). These HAV IgM and IgG immunoassays are easy to perform, rapid and sensitive and have been integrated into national guidance on the management of hepatitis A cases.

## Introduction

Hepatitis A virus (HAV) is a picornavirus causing faecal-orally transmitted acute liver disease, hepatitis A. The UK has a low annual incidence of infection [1]. In the absence of sustained endemic transmission and in the presence of high levels of hygiene and sanitation, the UK does not have universal hepatitis A childhood immunisation. Public Health England (PHE) recommends selective immunisation for to those who are at increased risk of infection including travellers to endemic areas, those at occupational risk, persons who inject drugs and men who have sex with men [2]. Susceptibility to HAV infection varies in the population; it is highest in those under 30 years of age, with >80% of such individuals being seronegative for antibody to HAV [3]. Seroprevalence increases with age and by the age of 60 more than three quarters of the population are seropositive from previous infection or immunisation [3]. There is potential for localised outbreaks in England and Wales, especially if primary schools are involved as young children are frequently implicated in spread due to variable levels of personal hygiene and high levels of susceptibility [4].

In England and Wales 290 confirmed cases were reported in 2016 [1]; of these 53.8% had a travel history. Where the source of the infection remained unknown these sporadic cases may have been person-to-person transmissions from sub-clinical undiagnosed infections in individuals from higher risk populations including those with undeclared recent travel to endemic countries, or direct infections from contaminated food. Asymptomatic infection in young children is often implicated in the spread of infection during extended outbreaks [4] and transmission of infection within affected households is relatively common [5]. When the index case is a child, serological screening of the household may indicate whether the child could have acquired their infection from an asymptomatic case in the household. Where this is found not to be the case, the possible acquisition from school contacts will direct public health intervention which may include mass immunisation in the school on the assumption that there is at least another case in the school and transmission has occurred in that setting. The primary tool for screening and diagnosis of acute HAV infection is serological detection of HAV immunoglobulin M (IgM) and immunoglobulin G (IgG). While venous blood sampling is acceptable to individuals who are ill and easier to perform in adults, this can be challenging when children are involved as they may need to attend a hospital for venesection. Oral fluid (ORF) sampling is much less invasive and has been used effectively in epidemiological studies and in public health surveillance including detecting serological responses to hepatitis A and other viruses, particularly in hard-to-reach populations [6, 7]. Self-sampling of the ORFs can easily be performed outside the healthcare setting with sampling kits being distributed to potential contacts and then returned by post directly to the laboratory for testing [8].

ORF assays for detection of HAV antibodies have been previously described [7–12], but to date none are commercially available. A single previous study has shown the value of ORF testing in identifying asymptomatic infections that are a potential transmission risk to close contacts [13]. The aim of the study presented here was to develop and validate the performance of an in-house modification of an accessible commercial assay for the clinical detection of HAV IgM and IgG antibodies in ORF specimens and to analyse the results from 1 year of testing in household contacts of confirmed hepatitis A cases in England and Wales.

## Methods

### HAV IgM and IgG controls

Five half log_10_ serial dilutions of pooled HAV IgM/IgG positive sera (Abbott Architect, Abbott, Maidenhead, UK) were prepared in normal human plasma (NHP, unreactive for anti-HAV). These and NHP were used as controls. To mimic ORF concentrations of immunoglobulins these controls were diluted 1/800 in viral transport media (VTM: phosphate buffered saline containing 10% calf serum, 0.5% gentamicin and 0.2% fungizone) prior to testing based on previous experience working with ORFs.

### ORF processing

ORF was eluted from Oracol swabs into 1 ml VTM by addition of VTM to the primary tube followed by slow withdrawal of the swab through the VTM with circular compression on the side of the tube. Following elution all sample extracts were stored at 2–8 °C prior to testing.

### ORF testing

HAV IgM and IgG were sought using reagents from a HAV IgM commercial kit (Fortress Diagnostics, UK), combined with antibody capture plates specific for either human IgM or IgG (Microimmune, UK). Briefly, 100 µl of ORF extracts, undiluted and further diluted 10-fold in VTM, or diluted plasma controls were added to either the anti-human IgM or IgG solid phase and incubated at 37 °C for 1 h. After washing five times (Fortress wash buffer) 100 µl of Fortress kit conjugate were added at 37 °C for 40 min. After washing as before, 50 µl of chromogen A and 50 µl of chromogen B (Fortress) were added, incubated for 15 min at 37 °C and the reaction then stopped with 50 µl stop solution. The plate was read at 450 nm with a reference filter of 630 nm. The cut-off value for a positive reaction was calculated by adding 0.1 to the mean optical density (OD) of three negative controls. Specimens with an OD/cut-off (OD/CO) ratio ⩾1.1 were considered reactive; those with a ratio ⩽0.9 were considered non-reactive. Samples with an OD/CO ratio between 0.91 and 1.09 were considered equivocal.

### Validation panel (*n* = 30 reactive and 40 non-reactive)

ORF samples were prospectively requested from 30 individuals with acute HAV infection confirmed at PHE (seropositive for HAV IgM and IgG with detectable HAV RNA viraemia). ORF samples were self-collected using the Oracol collection kit (Malvern Medical, UK) then posted directly to the Virus Reference Department (VRD) at PHE. Samples were kept at room temperature until processed as detailed above. To assess diagnostic specificity, 40 ORF known to be non-reactive by a previously available validated ORF assay [12] were tested. These 40 samples were retained from a previous HAV outbreak investigation within a school.

### Assessing the eluate immunoglobulin lower limit for valid detection of ORF antibody (limit of detection, LOD)

Due to self-sampling the quality of the ORF is variable. Measuring the total immunoglobulin level within ORF allows the quality of the sample to be assessed and validates a negative result. To assess the level of total immunoglobulin at which an acute case would not be identified, seven ORFs with sufficient volume for retesting from the acute HAV validation panel, previously analysed for total immunoglobulin, were randomly selected and subjected to half log_10_ serial dilutions in VTM before testing.

### Household contact panel (*n* = 280)

Following establishment of the assay, ORF specimens were prospectively sought by local Health Protection Teams (HPTs) between April 2015 and May 2016 from the household contacts of index cases whose plasma were confirmed to be HAV RNA positive. A household contact was defined as ‘A person living in the same household as the index case or regularly sharing food or toilet facilities with the index case during the infectious period, including extended family members and friends who frequently visit the household. This may also include those in shared accommodation (e.g. boarding schools) with shared kitchen and/or toilet facilities’ [14]. Household contacts of 72 index cases responded to the request and those with no history of HAV immunisation were offered post-exposure vaccine in line with current UK guidance. These contacts were invited to self-collect an ORF sample using an Oracol collection kit and post it to VRD for testing. Demographic and risk factor information (such as travel history and prior immunisation) were collected on the request form or via HPzone, a web-based surveillance and case management tool used by local HPTs within England.

### Statistical analysis

Sensitivity and specificity were calculated with exact 95% confidence intervals (95% CIs). To assess factors associated with transmission within a household the data were analysed at the household level. Transmission was defined as an individual other than the index case within the same household having HAV IgM and IgG reactivity. Univariable analysis was performed using Fisher's exact test, and multivariable analysis was performed using logistic regression (Stata v.13, StataCorp.) with household transmission (1 = yes/0 = no) as the dependent variable and independent variables of prior immunity in the household, index case travel history, index case age (<5, 5–10, 11–17, 18+), index case sex, presence of children in the household other than the index case (with three definitions of children as <5, <11, <18). Estimates were also adjusted for the total number of individuals tested in a household as this may affect the chance of detecting a transmission. Variables significant in univariable analyses were included in a multivariable analysis.

## Results

### Validation panel (*n* = 30)

Thirty samples were received for analysis, a median of 4.5 weeks post serum diagnosis (range −1 to 10 weeks). All but one ORF specimens were reactive for HAV IgM and IgG (Table 1) upon undiluted testing. Case 7 produced an equivocal IgM result and negative IgG result, with a total immunoglobulin level of 23 mg/l. This patient was known to have HAV IgM and IgG reactivity in their serum sample and when the ORF was tested at 1/10 dilution in VTM significant HAV IgM and IgG reactivity was observed. The 29 other samples were also tested at a 1/10 dilution in VTM (Table 1) with four becoming negative and one equivocal by IgM and two negative by IgG. The sensitivity for IgG and IgM was therefore 96.7% (29/30, 95% CI: 82.8–99.9%) for undiluted ORF testing, 83.3% (25/30, 95% CI: 65.3–94.4%) for IgG diluted, and 93.3% (28/30, 95% CI: 77.9–99.2%) for IgM diluted. All samples were detected either at neat or 1/10 dilution testing. All 40 non-reactive ORF samples were found to be unreactive for HAV IgM and IgG giving a specificity for IgG and IgM of 100% (95% CI: 91.2–100%). To assess the reproducibility of the assays 40 samples were re-tested by another operator with different component lot numbers giving a concordance of 100% for the IgM assay and 97.5% for the IgG assay (kappa value 0.9; 95% CI: 0.753–1). Repeatability testing was performed by repeat testing 40 samples by the same operator with the same component lot numbers, giving a concordance of 100% for both assays (kappa value 1; 95% CI: 1–1).

**Table 1.** Results of HAV IgM and IgG testing on 30 ORF samples from individuals with serologically confirmed HAV infection and detectable HAV RNA

Case	Age	Sex	IgM OD/CO		IgG OD/CO		Weeks post serum sample bleed	Total IgG (mg/l)
			Undiluted	1/10 dilution	Undiluted	1/10 dilution		
1	1	M	18.17	9.93	26.82	21.07	6	8.3
2	5	F	16.28	6.74	24.43	18.37	6	1.9
3	22	F	15.48	2.64	19.13	14.88	1	1.9
4	47	M	13.06	1.36	21.03	18.08	2	8.4
5	7	F	2.82	0.05^a^	9	0.016^a^	3	0.3
6	15	M	1.59	0.17^a^	20.25	20.1	10	4.3
7	33	M	0.92	14.36	0.36	12.16	Unknown	23
8	21	F	18.81	16.09	21.39	22.42	6	17
9	14	F	19.96	9.43	17.99	17.66	2	13
10	5	F	18.52	7.62	16.7	10.32	1	3.2
11	2	M	26.58	26.62	1.43	0.64	1	2.2
12	6	F	7.74	1.29	20.2	20.88	Unknown	4.6
13	4	M	13.39	2.01	22.84	13.32	4	1.4
14	5	F	8.5	1.04	19.3	9.67	4	1.4
15	7	F	16.74	4.03	21.67	18.93	1	4.7
16	15	M	23.5	16.92	14.68	16.58	1	14
17	23	M	22.65	14.42	18.07	18.76	6	>25
18	63	M	5.31	1.1	18.18	17.47	1 week prior^b^	3.8
19	5	F	7.45	1.77	11.88	10.82	Unknown	1.3
20	20	F	30.21	10.59	32.16	31.38	Unknown	8.9
21	70	F	25.57	10.68	29.22	28.5	6	14
22	16	F	21.16	4.9	28.59	29.31	4	7
23	14	M	25.15	14.88	27.47	27.08	5	>25
24	17	F	30.98	15.88	26.93	27.26	6	11
25	3	M	2.91	5.17	14.64	23.28	6	2.3
26	9	M	4.75	0.13^a^	19.86	3.3	5	0.2
27	11	M	12.1	5.44	23	23.74	4	5
28	8	M	24.88	13.5	23.16	23.08	6	6.3
29	8	F	19.63	10.2	18.77	19.88	6	7.2
30	6	F	6.51	0.74^a^	22.04	20.17	3	4.1

OD/CO ratio ⩾1.1 indicates reactivity.

a Indicates a sample which became non-reactive on dilution.

b Patient was identified through ORF testing after contact with a known case – HAV RNA was detectable in a serum sample taken 1 week post ORF sampling.

### Assessment of the lower LOD of total anti-IgG in cases of acute HAV infection

The LOD for a sample was defined as the lowest level of total anti-IgG where both the HAV IgM and IgG both remained detected at S/CO ratios ⩾1.1. HAV IgM and IgG reactivity were reliably detected in ORF down to total immunoglobulin concentrations of 0.4 and 0.03 mg/l respectively (Table 2).

**Table 2.** Lower LOD of total anti-IgG in ORFs taken from seven individuals serologically confirmed HAV infection and detectable HAV RNA

Sample	Lowest total anti-Ig level (mg/l) with detectable HAV IgM	OD/CO	Lowest total anti-Ig level (mg/l) with detectable HAV IgG	OD/CO
1	0.30	2.09	0.030	2.57
2	0.02	1.12	0.023	1.97
3	0.14	1.53	0.005	2.22
4	0.43	3.12	0.043	4.19
5	0.11	1.10	0.011	1.87
6	0.02	1.36	0.022	4.09
7	0.02	1.16	0.007	1.1

The table details the lowest level of total anti-IgG in each sample where the HAV IgG or IgM gave an OD/CO ratio of ⩾1.1.

### Household contact panel (*n* = 280)

**Fig. 1.**
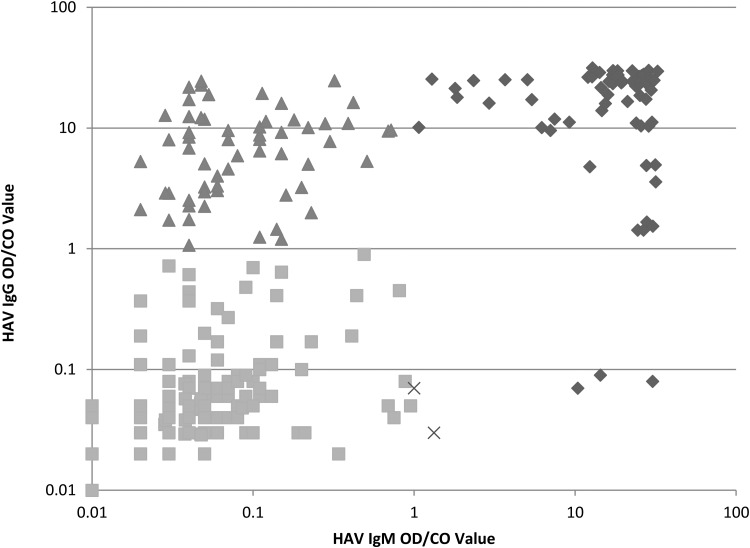
The relationship between HAV IgM and IgG reactivity in household contacts. Results of enzyme-linked immunoassay HAV IgM and IgG testing of *n* = 280 contacts. Values are the OD/CO value, plotted on a log_10_ scale. Diamonds denote individuals with recent HAV infection (the three samples in lower right corner demonstrate incubating HAV infection, with IgM reactivity in the absence of IgG), triangles those with past infection or immunisation (detectable HAV IgG in the absence of IgM) and squares those with no evidence of recent or past HAV infection. Crosses indicate the two individuals with low level IgM reactivity of unknown significance.

**Fig. 2.**
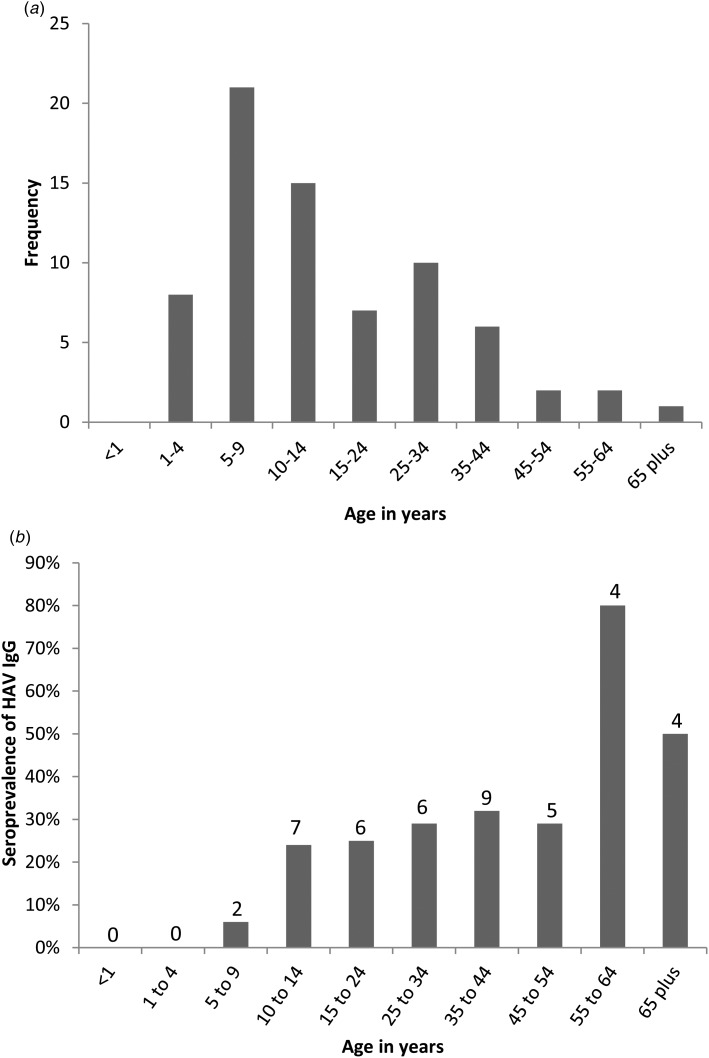
(a) Age distribution of the 72 HAV infected index cases. (b) Age specific prevalence of HAV-IgG in the household contact cohort. Numbers above the bars indicate the number of individuals within that age group with HAV-IgG reactivity.

Two hundred and eighty household contacts from 72 households associated with index cases responded and provided an ORF sample. A median of three household contacts were tested per index case (range 1–12). The age distribution of the 72 index cases is shown in Figure 2a, with a median age of 11 years (range 2–67 years, interquartile range (IQR): 7–26 years), and a mean age of 18 years. The serological reactivity of 68 of the 280 ORFs (24.3%) indicated recent acute HAV infection (Fig. 1) revealing that 35 of the 72 households had recent HAV transmission within the household. The clear majority (92.6%; 63/68) of IgM reactive ORF samples came from individuals aged 14 years or less. Three individuals, two children and one adult were found to be in the incubation phase, with significant HAV IgM levels with no concurrent IgG reactivity.

Of this cohort 60 (21.4%) individuals had isolated IgG ORF reactivity from past HAV infection or previous HAV immunisation. The majority (75%; 45/60) were adults aged ⩾18 years of age, with the age specific prevalence increasing with age (Fig. 2b). Just over half (53.6%; 150/280) of all ORFs contained no detectable antibody to HAV. Two ORFs (0.7%) had isolated low level IgM reactivity of unknown significance.

### Factors associated with household transmission

**Fig. 3.**
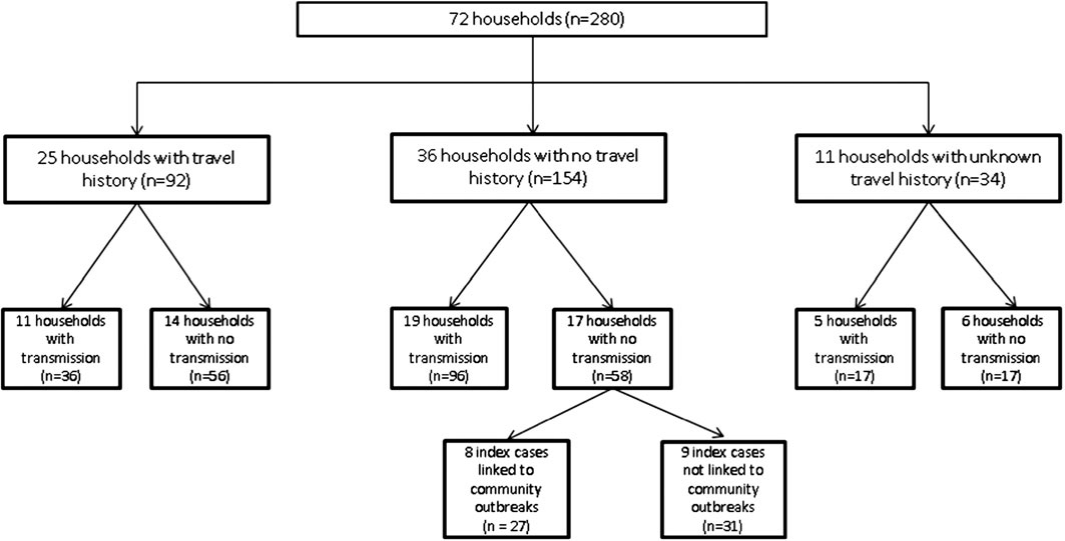
Flow chart of 72 households participating in the study. Numbers in brackets denote number of household contacts within that grouping.

Twenty-five of the households (34.7%) reported recent travel outside the UK, 36 (50%) households had no recent history of travel outside the UK and the remaining 11 households (16.9%) provided no travel risk factor information (Fig. 3). Of the travel-associated households 44% (11/25 cases) had evidence of transmission of HAV within their homes; similarly, 52.8% (19/36 cases) of those households who reported no travel outside the UK during the incubation period also had evidence of recent transmission within the home.

After multivariable analysis of the data the only significant factor for transmission within the household was the presence of children under the age of 11 (odds ratio = 9.84 (95% CI 2.74–35.37), *P*-value <0.001; Table 3). Evidence of prior immunity within the household and presence of additional children of any age were significant factors on univariable analysis (*P* = 0.04 and 0.012 respectively); however, these became insignificant when adjusted for the presence of children aged <11 years. There was no evidence of effect modification between the presence of children and evidence of prior immunity (*P* = 0.58).

**Table 3.** Univariable and multivariable analysis of relationship between factors and household transmission

Factor	Level	Transmission (*n*/*N*, %)	Univariable odds ratio (OR) (95% CI)	Exact *P*-value	Multivariable OR^a^ (95% CI)	*P*-value
Travel	No	19/36 (53)	Baseline	0.61		
	Yes	11/25 (44)	0.70 (0.25–1.96)			
Gender	Female	15/33 (45)	Baseline	0.60		
	Male	15/27 (56)	1.50 (0.54–4.17)			
Prior immunity in household	No	23/38 (61)	Baseline	0.04	Baseline	0.15
	Yes	12/34 (35)	0.36 (0.14–0.93)		0.43 (0.14–1.34)	
Index age	<5	4/8 (50)	1.17 (0.24–5.70)	0.23		
	5–11	12/2 (46)	Baseline			
	12–17	3/12 (25)	0.39 (0.09–1.77)			
	18+	16/26 (62)	1.87 (0.62–5.63)			
Other children in house	No	0/7 (0)	Baseline	0.012		
	Yes	35/65 (54)	Inf (−)			
Other children <11 in house	No	6/31 (19)	Baseline	<0.001	Baseline	<0.001
	Yes	29/41 (71)	10.07 (3.3–30.8)		9.84 (2.74–35.37)	
Other children <5 in house	No	24/55 (44)	Baseline	0.17		
	Yes	11/17 (65)	2.36 (0.77–7.32)			

a Model just includes past history of exposure in the household, other children aged <11 in the household and total tests done.

The likely source of initial infection within household could be inferred by preceding travel history. In the 25 households where members provided a travel history, 21 persons (84%), had travelled to countries where pre-travel HAV immunisation is recommended. Often travellers who are going ‘home’ to visit friends and family do not perceive that they are at risk of infection [15]. Of the 36 households with no travel history 19 households had another member with antibody evidence of recent infection, suggesting transmission within the household between the index case and other family members. There were 17 households in which cases arose in the absence of any travel history and demonstrable household transmission. Eight cases were phylogenetically associated (data not shown) with contemporaneous community outbreaks leaving nine sporadic cases. Of these nine cases four individuals had sequences which had been seen before from HAV enhanced molecular surveillance in England and Wales. Five had unique sequences, which could have been the result of contaminated food products or importation from unidentified travel-related cases.

## Discussion

The assays described here appear sensitive, robust and able to detect antibody in ORF samples containing low immunoglobulin levels. HAV IgM and IgG were reliably detected in samples with immunoglobulin levels of ⩽0.4 mg/l demonstrating that these assays are likely to detect recent HAV infections even in poor quality samples containing low levels of immunoglobulin. Furthermore, ORFs collected later from individuals with acute HAV infections showed that recent HAV infection could be diagnosed by ORF swabbing up to 10 weeks after clinical onset of disease.

One concern was an apparent single ORF (case 7 from the validation panel) which produced a discrepancy between ORF and serology. When tested undiluted the ORF was equivocal for HAV IgM and negative for HAV IgG, however, testing at one in 10 dilution led to a strong reactivity in both assays. The total immunoglobulin content in the sample was unremarkable (23 mg/l). A further sample in the validation panel (case 25) also showed increased reactivity when tested following dilution; however this did not affect the clinical interpretation of the result. Testing of the validation panel at a 1/10 dilution showed loss of HAV IgM and/or IgG reactivity in some samples which were taken ⩾6 weeks after serum sample bleed or where the total anti-IgG was <0.4 mg/l. The apparent inhibitory effect observed in the ORF of case 7 is likely to be a rare occurrence as this phenomenon was only observed in two of the 280 household contacts tested, where unreactive ORF samples became weakly reactive upon dilution. This effect of increasing reactivity in diluted ORF was seen in a small proportion of household contact samples, however did not lead to a discrepancy in clinical interpretation between the neat and diluted sample. The mechanism by which an ORF sample may mask serologic reactivity remains unclear. At present this can be avoided by testing the ORF samples both undiluted and at a further 10-fold dilution in VTM thus mitigating the risk of false negative results. Clearly this merits further investigation.

Of the 280 household-contact ORF samples 68 (24.3%) provided evidence of recent HAV infection; only six of 68 individuals went on to develop symptomatic disease. Importantly this clearly demonstrates a high level of undiagnosed subclinical HAV infections equivalent to an additional 0.79 cases per clinical infection and results in nearly doubling the incidence of HAV infection within this cohort. This is not surprising given the majority (55/68; 80.9%) of the recent infections were in children less than 10 years of age. Of note, there were two adults who were identified as having recent infections who were asymptomatic at the time of screening and who remained asymptomatic thereafter. While it is known that the likelihood of symptomatic infection increases with age, cases of asymptomatic HAV infection in adults have been documented [16].

This study demonstrates that ongoing person to person transmission of HAV is occurring in the community via households with asymptomatic children being the main vector. This is supported by the multivariable analysis identifying children aged 11 years and under as being significantly associated with household transmission. The findings of subclinical infection in households where no travel history was identified support the current public health guidance recommendations to screen all household contacts of index cases in children.

Two household contacts had low level IgM reactivity of unknown significance in the absence of HAV IgG reactivity. Both of these were from children (aged 1 and 7 years) and this HAV IgM reactivity could have been due to incubating HAV infection. Without access to follow-up samples for these patients we are unable to ascertain if these individuals were sampled early in the incubation period, or whether this was non-specific reactivity.

The studies described here show the benefit of ORF testing as a socially acceptable and non-invasive approach which has many advantages when applied to those populations more averse to venesection. These studies have also demonstrated that the transmission of HAV within the household setting is a common occurrence, particularly in the presence of young children. The HAV IgM and IgG immunoassays described here are easy to perform, rapid and sensitive; detecting HAV antibodies present in ORF even in poorly taken swabs. Following this study ORF testing for HAV has become embedded in PHE guidance [14], as due to the rapid nature of these assays results can be passed to public health officials within a few hours of sample receipt in the laboratory.
